# Retenção das Habilidades de Ressuscitação Cardiopulmonar nos Estudantes de Medicina

**DOI:** 10.36660/abc.20200546

**Published:** 2021-08-04

**Authors:** Miguel Antônio Moretti, Adriana de Oliveira Camboim, Caroline Awoki Ferrandez, Isabela Corralo Ramos, Iaggo Bemini Costa, Juliana Seidler Canonaco, Vanessa Lopes Mathia, João Fernando Monteiro Ferreira, Antonio Carlos Palandri Chagas

**Affiliations:** Faculdade de Medicina Fundação do ABC Santo André SP Brasil Faculdade de Medicina da Fundação do ABC – Cardiologia,1 Santo André, SP – Brasil

**Keywords:** Reanimação Cardiopulmonar, Mortalidade, Parada Cardíaca, Estudantes de Medicina, Educação, Aprendizagem, Habilidade

## Abstract

**Fundamento:**

A redução da mortalidade e das sequelas de uma vítima de parada cardíaca depende de um atendimento eficaz, rápido e iniciado o mais precocemente possível. O suporte básico de vida (SBV) compreende uma série de etapas que podem ser iniciadas fora do ambiente hospitalar, e ensinadas para qualquer pessoa em cursos específicos. Porém, é importante que o socorrista retenha o conhecimento e as habilidades, pois nunca se sabe quando será necessário realizar uma ressuscitação cardiopulmonar (RCP). Entretanto, estudos mostram que existe uma perda das habilidades em executar uma RCP já com 30 dias após o treinamento, com variações segundo algumas características das pessoas e da atividade profissional.

**Objetivo:**

Avaliar se os estudantes de medicina são capazes de reter as habilidades por mais de seis meses.

**Métodos:**

Estudo prospectivo, caso controle, observacional. Estudantes de medicina realizaram um curso sobre morte súbita e parada cardíaca de 40 horas. A avaliação das habilidades foi realizada imediatamente após o curso e seis meses depois. Foram comparadas as notas individuais entre dois momentos, foi avaliada a porcentagem de acerto em cada etapa e uma análise global do atendimento foi classificado como ótimo, bom e ruim. Os avaliadores e critérios foram os mesmos nos dois momentos. Os dados foram analisados pelos teste-t pareado e teste de McNemar, onde para um nível de confiança de 95% o critério para significância foi p < 0,05.

**Resultados:**

Cinquenta estudantes (27 do sexo feminino) do primeiro ano, com idade entre 18 e 24 anos (média 21), realizaram o curso. O número de etapas cumpridas de forma correta após seis meses foi significativamente menor que logo após o curso (10,8 vs 12,5 p < 0,001). O sexo e idade não interferiram nos resultados. A qualidade global foi considerada ótima em 78% dos atendimentos realizados logo após o curso, significativamente, maior que os 40% após seis meses (p < 0,01). Após seis meses, maior número de erros foi observado nas etapas relacionadas às habilidades mais práticas (como posicionamento das mãos).

**Conclusão:**

Seis meses após o curso observamos uma perda significativa das habilidades, entre estudantes de medicina, prejudicando a eficácia global do atendimento.

## Introdução

As doenças isquêmicas do coração são as principais causas de morte por doenças cardiovasculares,^1^ morte súbita (MS), e de parada cardiorrespiratória (PCR)^2^ na população brasileira. A tentativa de reversão da PCR é feita por manobras de ressuscitação cardiopulmonar (RCP) e a redução da mortalidade e de sequelas de uma vítima de PCR depende de um atendimento eficaz, rápido e mais precoce possível, de preferência no local de sua ocorrência.^2 , 3^

O suporte básico de vida (SBV) compreende uma série de etapas que podem ser iniciadas fora do ambiente hospitalar.^4 , 5^ Em um curso de SBV, as técnicas de RCP abordadas vão desde o reconhecimento precoce da parada cardíaca, do início imediato das manobras de compressão torácica e ventilação, até o uso do desfibrilador externo automático (DEA), e podem sem ensinadas para qualquer pessoa.^5 - 7^ Como a maioria dos eventos de PCR ocorre fora do ambiente hospitalar, é importante que a população saiba executar as técnicas de RCP,^8 - 10^ mesmo que as pessoas achem que somente os profissionais da área da saúde sejam capazes de agir adequadamente em emergências.

Contudo, não basta apenas um treinamento adequado; é importante que o socorrista retenha o conhecimento e as habilidades para manter a eficiência da RCP, pois nunca se sabe quando será necessário colocar esses conhecimentos em prática. Diversos estudos foram realizados para avaliar a capacidade de retenção dessas informações e habilidades ao longo do tempo.^11 , 12^ No entanto, eles não são unânimes em apontar as principais causas da queda na retenção e nem em quanto tempo isso acontece. Isso dificulta, por exemplo, estabelecer com qual periodicidade deve-se realizar o retreinamento.

Estudos mostram que, mesmo entre profissionais da saúde existe uma perda das habilidades em executar uma RCP e apontam como causas desse insucesso o treinamento insuficiente e/ou falta de retenção de habilidades.^13^ Mesmo esses profissionais, às vezes, passam muito tempo sem utilizar esses conhecimentos. Smith et al.,^11^ demonstraram que as habilidades psicomotoras na RCP têm uma queda após 10 semanas em estudantes de graduação em enfermagem, e outros estudos destacam que as habilidades práticas se deterioraram já a partir dos primeiros 30 dias após o curso de suporte de vida avançado (SVA) e essa perda vai se acentuando até se estabilizar ao final de um ano.^14^ Estudo publicado em 2014 ressalta uma redução na retenção de habilidades entre estudantes de medicina após um e dois anos do curso de RCP.^15 , 16^

Como qualquer pessoa, os estudantes de medicina também estão sujeitos a testemunharem eventos de MS ou de PCR fora do ambiente hospitalar, o que já justificaria um treinamento sobre manobras de RCP.^17 , 18^ Desde seu ingresso na faculdade, logo nos primeiros meses, os estudantes já são cobrados pela sociedade e por eles mesmos a agirem como médicos, esperando que tenham as mesmas habilidades de um profissional.^19 , 20^ Por isso, defendemos que o treinamento para o atendimento à PCR faça parte do currículo do curso de medicina,^12^ onde o SBV seria ensinado logo no primeiro ano e o SVA mais no final do curso quando o estudante já possui uma carga maior de conhecimento, habilidades e logo estará trabalhando na assistência a pessoas. Entendemos que os cursos do *American Heart Association* (AHA) – por exemplo, o *Basic Life Support* (BLS) e o *Heart Saver* – e da Sociedade Brasileira de Cardiologia (Treinamento de Emergências Cardiovasculares Básico – TECA B) atendem as necessidades do treinamento para profissionais da saúde já formados ou para uma população leiga. Mas, para os estudantes do primeiro ano de medicina, o curso básico deveria incluir não somente o treinamento de habilidades, mas também proporcionar uma base teórica mais ampla, para que o aprendizado seja mais fácil e a retenção das habilidades aprendidas de maneira mais duradoura^12 , 17^ .

Nossa hipótese é de que a perda de conhecimento e de habilidades para realizar as manobras de RCP já está presente entre estudantes de medicina seis meses após um treinamento de SBV, mesmo no caso de cursos mais elaborados e de maior duração.

## Metodologia

Cinquenta estudantes (27 do sexo feminino) do primeiro ano da graduação do curso de medicina, com idade entre 18 e 24 anos (média 21), realizaram o curso de “Morte Súbita e Ressuscitação Cardiopulmonar”. Esse é um curso optativo dentro da grade curricular da graduação com carga horária de 32 horas-aula, sendo cerca de 30% de aulas teóricas e 70% de aulas práticas, com ênfase no desenvolvimento de habilidades e simulações de situações de MS e/ou PCR.

Além de um conteúdo teórico sobre a história, epidemiologia e fisiopatologia da MS, o curso também contempla uma parte prática onde os alunos são treinados para executar o atendimento a uma situação de PCR. Durante as aulas práticas são desenvolvidas e treinadas habilidades como reconhecer os sinais de uma PCR e quais atitudes tomar – verificar a segurança do local, saber quem e como chamar ajuda, como executar uma compressão torácica eficiente (força, frequência, profundidade, localização e posicionamento das mãos), como executar uma ventilação segura e eficaz utilizando dispositivos adequados, identificar a necessidade do uso de um DEA, como utilizá-lo e como dar sequência ou decidir pela interrupção do atendimento. Esse treinamento prático é baseado nos cursos de padronização do atendimento da PCR recomendado pela AHA^16^ (o BLS) e pela Sociedade Brasileira de Cardiologia (o TECA B).^5^

Após o curso, os estudantes tiveram os conhecimentos e as habilidades para o atendimento de uma PCR avaliados em ambiente extra-hospitalar. Para tanto, foi utilizada uma ficha padronizada com etapas que deveriam ser cumpridas de forma correta ( Quadro 1 ).

**Quadro 1:** – Formulário usado para avaliar conhecimento e habilidades adquiridos no curso “Morte Súbita e Ressuscitação Cardiopulmonar” aplicado imediatamente após e seis meses depois do curso

ETAPAS A SEREM EXECUTADAS APÓS IDENTIFICAR UM INDIVÍDUO INCONSCIENTE, QUE NÃO SE MEXE E NÃO RESPIRA
Avaliar a segurança do local e da execução de atendimento
Solicitar ajuda a outra pessoa de forma clara e objetiva, dizendo o que é para fazer
Definir para quem ligar - 192/193
Solicitar o desfibrilador externo automático (DEA)
Colocar vítima na posição correta e verificar pulso e respiração
Iniciar compressões torácicas de imediato
Colocar as mãos na posição correta sobre o esterno
Exercer cada compressão na profundidade correta (4-5 cm)
Exercer compressões na frequência correta (100 vezes por minuto)
Realizar ventilação somente com dispositivo de proteção
Manter os 5 ciclos de 30 compressões e 2 ventilações antes de reavaliar a vítima
Não interromper a ressuscitação cardiopulmonar na chegada do DEA
Utilizar o DEA de acordo com instruções do aparelho
Retomar as manobras de ressuscitação (RCP) após uso do DEA se necessário

Cada etapa realizada pelo aluno foi avaliada como cumprida ou não (SIM se realizou de forma correta e NÃO se não realizou de forma correta, ou se não realizou). Ao final, o atendimento como um todo (atendimento global) foi avaliado em: ótimo, bom ou ruim. Para um ótimo desempenho, o aluno poderia cometer até dois erros (o que estaria acima dos 84% de acertos que é exigido pelo AHA^16^ ); para um bom desempenho, três ou quatro erros (mais de 70% de acertos); e ruim se cometesse mais de quatro erros (menos de 70% de acertos). Setenta por cento de acertos é o mínimo esperado para o aluno ser aprovado no curso de acordo com os critérios da faculdade.

Após seis meses do curso, os estudantes foram submetidos a uma reavaliação, onde realizaram um atendimento simulado de PCR em manequim, sem agendamento prévio. Foi utilizada a mesma ficha de avaliação e os mesmos critérios (mesmos instrutores/professores). O atendimento global também foi classificado.

Todos os alunos assinaram termo de consentimento livre e esclarecido aceitando participar do estudo. O estudo, a aprovação pelo comitê de ética e o termo de consentimento livre e esclarecido foram aprovados e registrados na Plataforma Brasil (CAAE: 81721317.7.0000.0082).

## Análise Estatística

Trata-se de um estudo prospectivo, onde cada indivíduo foi controle dele mesmo, em uma amostra por conveniência. Quando se comparou etapa por etapa, os resultados foram avaliados pelo teste de McNemar para as variáveis categóricas (apresentadas em valores absolutos e em porcentagem). As médias e desvios padrões das variáveis contínuas foram comparadas pelo teste t pareado usando o programa Excel da Microsoft Office 365^™^, após confirmação da normalidade pelo teste de Shapiro-Wilk. O nível de confiança de 95% foi calculado e valor de p menor que 0,05 foi estabelecido para significância estatística.

## Resultados

**Figura 1:**
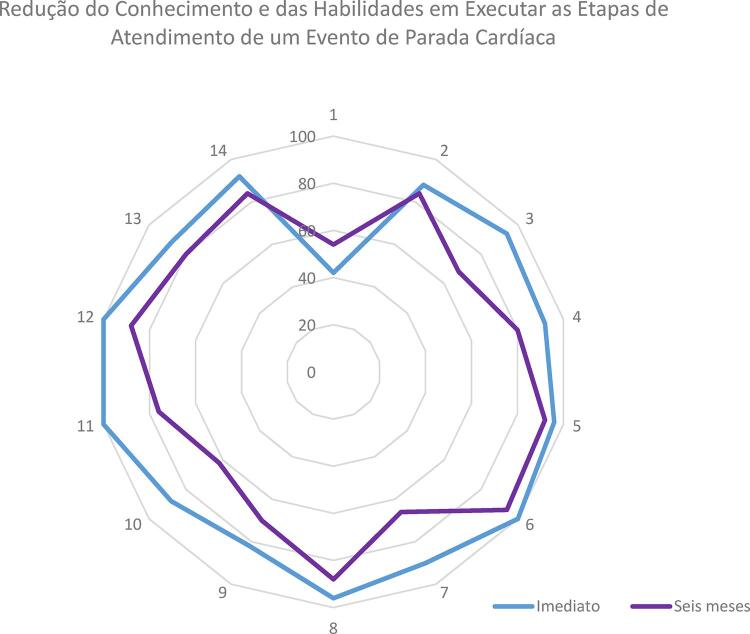
– *Porcentagem de alunos que executaram de forma correta as etapas do atendimento, imediatamente após o curso e seis meses depois. A numeração corresponde à adotada na Tabela 1.*

Todos os alunos foram avaliados logo após o curso e seis meses depois. Das 14 etapas avaliadas, os estudantes cumpriram de forma correta, em média, 12,5 etapas logo após o curso e em média 10,8 etapas na avaliação após seis meses. Houve uma diferença significativa (p < 0,001), demonstrando uma perda na retenção do conhecimento e das habilidades após o período de seis meses. Também observamos que, apesar de os homens terem uma média de idade maior que as mulheres (21,7 anos vs. 20,2 anos – p = 0,006), a idade e o sexo dos alunos não interferiram de forma independente no resultado das avaliações. Os homens tiveram uma redução de acertos (12,8 acertos antes e 10,9 acertos depois) significativa (p = 0,003), assim como as mulheres (12,2 acertos antes e 10,7 acertos depois) significativa (p = 0,013). Na avalição do atendimento global, também foi observado uma piora significativa na qualidade do atendimento, com 39 (78%) atendimentos considerados ótimos logo após o curso e 20 (40%) atendimentos considerados ótimos seis meses após (p < 0,01). As porcentagens de alunos que executaram de forma correta cada etapa avaliada logo após o curso e na reavaliação após seis meses encontram-se na Tabela 1 e Figura 1 .

**Tabela 1:** – Resultado da avaliação do conhecimento adquirido no curso “Morte Súbita e Ressuscitação Cardiopulmonar”, por número e porcentagem de alunos que cumpriram cada etapa, logo após o curso e seis meses depois

Etapa	Porcentagem de alunos que realizaram de forma correta		P*

	Imediato (N)	Seis meses (N)	
1 - Avaliar a segurança	42 (21)	54 (27)	0.307
2 - Solicitar ajuda	88 (44)	84 (42)	0.683
3 - Definir para quem ligar	94 (47)	68 (34)	**0.002**
4 - Solicitar o DEA	92 (46)	80 (40)	0.181
5 - Verificar pulso e ventilação	96 (48)	92 (46)	0.683
6 - Iniciar compressões torácicas	100 (50)	94 (47)	0.248
7 - Colocar as mãos na posição correta	90 (45)	66 (33)	**0.010**
8 - Profundidade correta da compressão	96 (48)	88 (44)	0.289
9 - Frequência correta das compressões	82 (41)	70 (35)	0.264
10 - Realizar ventilação com dispositivo de proteção	88 (44)	62 (31)	**0.010**
11 - Manter os 5 ciclos de 30:2 antes de reavaliar	100 (50)	76 (38)	**0.002**
12 - Não interromper a RCP na chegada do DEA	100 (50)	88 (44)	**0.041**
13 - Utilização do DEA de acordo com instruções	88(44)	80 (40)	0.387
14 - Retomar RCP após uso do DEA se necessário	92(46)	84 (42)	0.387

* valor de p calculado pelo teste de McNemar (bicaudal); RCP: ressuscitação cardiopulmonar; DEA: desfibrilador externo automático.

## Discussão

O nosso estudo demonstrou uma redução significativa (p < 0,01) das habilidades para o atendimento da PCR. Os alunos completaram corretamente, em média, 12,5 etapas do atendimento logo após o curso contra 10,8 etapas corretas após seis meses. Enquanto 39 estudantes (78%) completaram corretamente mais de 12 das 14 etapas do atendimento simulado logo após o curso, apenas 20 (40%) atingiram o mesmo escore após seis meses do curso.

Nós avaliamos somente o aspecto referente às habilidades necessárias para um atendimento adequado e correto da PCR e observamos ao final do curso que os alunos demonstraram ter aprendido de forma satisfatória essas habilidades. Apenas a primeira etapa, onde eles deveriam checar a segurança antes de iniciar o atendimento, não foi realizada de forma correta pela maioria dos estudantes (42%). Quando questionados sobre isso, os estudantes alegaram que como estavam em uma sala de aula, atendendo um manequim estático e num ambiente já sabidamente seguro eles se esqueciam de checar a segurança. Outra justificativa foi a ansiedade por iniciar rapidamente o atendimento. Mesmo assim, o aproveitamento demonstrado ao final do curso foi adequado ao exigido pela AHA, que deve ser maior que 84%.^16^ Observou-se também que idade e sexo não interferiram nas notas dos alunos ao final do curso, reforçando que o curso foi um fator importante no aprendizado.

Um problema frequentemente relatado nos estudos sobre ensino do atendimento à PCR é como manter a retenção do que foi ensinado. Uma metanálise^14^ sobre o assunto deixa claro que as habilidades se perdem ao longo do tempo se não praticadas ou exercitadas, iniciando-se com poucas semanas e atingindo um ponto mais elevado de perda com cerca de nove meses há um ano. Esses estudos mostram que o tempo decorrido desde o último treinamento é diretamente proporcional ao grau de perda das habilidades e do conhecimento necessário para atender uma PCR.^14^

Em nosso estudo, observamos que essa perda também acontece de forma significativa com os estudantes de medicina do primeiro ano do curso, com uma perda de aproximadamente 15% na nota média da prova de habilidades, o que impactou numa perda de cerca de 50% na qualidade do atendimento global. Não esperávamos que a perda de retenção fosse diferente entre os estudantes de medicina, pois outros estudos com profissionais da saúde ou estudantes demonstram a ocorrência dessa perda,^13^ e por sabermos que os estudantes não praticam ou atendem com frequência eventos de PCR. Na realidade, seria necessário um estudo comparando essa perda entre estudantes de medicina e outros profissionais.

Na população estudada, foi nítida a redução das habilidades. Observamos que as perdas mais significativas foram nas etapas onde as habilidades práticas requerem agilidade e mais atenção. Vários outros estudos também mostraram que essas etapas foram mais esquecidas ou realizadas de forma inadequada.^21 , 22^ A não realização adequada dessas etapas (definir para quem ligar; colocar as mãos de forma correta e no local certo; realizar ventilação com dispositivo de proteção; manter os 5 ciclos e não interromper as manobras com a chegada do DEA) pode favorecer a ocorrência de eventos adversos ou complicações decorrentes do atendimento como, por exemplo, fratura de costelas e compressões ineficientes (que não geram circulação eficiente).^23^

Ainda, nem idade nem sexo interferiu na perda da retenção de conhecimento e habilidades por nossos estudantes, e o tempo parece ter sido o único fator responsável pela perda de retenção. Isso é reforçado pelo fato de que nenhum dos estudantes atendeu eventos de PCR ou auxiliou na administração de cursos de RCP.

Uma estratégia para manter a retenção das habilidades, conforme proposto em outras publicações,^17 , 24^ seria um retreinamento periódico como, por exemplo, e-learning. Porém, uma questão ainda não respondida seria qual o intervalo e periodicidade do treinamento, qual tipo de treinamento e para qual tipo de população (profissionais da saúde ou não, que atuam em locais com elevada ocorrência de eventos de PCR). Outra questão é se o curso inicial deveria ser mais completo ou mais curto, particularmente para estudantes de medicina. Essa questão seria respondida por um estudo comparativo entre os dois métodos.

A limitação desse estudo está no número de alunos avaliados e por ter sido, apesar de prospectivo, uma observação em um único centro e após um único treinamento. Ruijter et al.,^15^ mostraram uma redução significativa das habilidades adquiridas por 120 alunos de medicina um e dois anos após um curso de SBV, semelhante ao que demonstramos. Assim, embora o objetivo tenha sido analisar a retenção das habilidades, nossos resultados foram muito semelhantes aos de outros estudos, essa limitação não compromete a importância do estudo. Outra limitação para aprofundarmos a discussão sobre a retenção e sobre qual seria o melhor tipo de curso é que os conhecimentos teóricos não foram avaliados, apesar de alguns estudos mostrarem que as perdas se dão mais nas habilidades práticas.^11^ Embora os alunos soubessem que seriam reavaliados em algum momento depois do curso, isso parece não ter comprometido o resultado pois muitos deles haviam se esquecido que seriam reavaliados, pois na realidade já haviam sido aprovados no curso. Tal fato, porém, não minimiza a necessidade de os cursos de retreinamento serem mais frequentes, para tentar reduzir a perda das habilidades.

## Conclusão

Seis meses após um curso com treinamento de atendimento simulado a vítimas de MS ou PCR, observamos uma perda significativa das habilidades em estudantes do primeiro ano da graduação em medicina, assim como observado na população que não é da área da saúde. Essa perda foi relacionada com o período em que os estudantes ficaram sem praticar ou sem revisitar as técnicas e o conhecimento de se realizar as etapas da RCP corretamente, e isso prejudica a eficácia do atendimento. Um curso mais robusto parece melhorar o aprendizado, mas ainda assim não melhora a retenção das habilidades. A avaliação do aprendizado e da retenção poderia ser complementada com estudos envolvendo retreinamento e resultado clínico, ou seja, mostrar que treinar e retreinar melhora não só habilidades, mas que também pode salvar mais vidas.
