# Supplementary material for: Retenção das Habilidades de Ressuscitação Cardiopulmonar nos Estudantes de Medicina
Source: Arq Bras Cardiol. 2021 Aug 4;117(5):1030–5. [Article in Portuguese] doi: 10.36660/abc.20200546 (PMC8682095; doi:10.36660/abc.20200546)
Supplement: Translation

# Translation: Retention of Cardiopulmonary Resuscitation Skills in Medical Students

**Authors:** Miguel Antônio Moretti, Adriana de Oliveira Camboim, Caroline Awoki Ferrandez, Isabela Corralo Ramos, Iaggo Bemini Costa, Juliana Seidler Canonaco, Vanessa Lopes Mathia, João Fernando Monteiro Ferreira, Antonio Carlos Palandri Chagas

**Affiliations:** 1 Brasil Faculdade de Medicina da Fundação do ABC – Cardiologia, Santo André, SP – Brasil

**Keywords:** Cardiopulmonary Resuscitation, Mortality, Heart Arrest, Medical Students, Education, Learning, Hability

## Abstract

**Background:**

Reduction of mortality and sequelae of cardiac arrest depends on an effective and fast intervention, started as soon as possible. Basic life support involves a series of steps that may be initiated out of the hospital setting and taught to any person in specific courses. However, it is important that the rescuers retain the knowledge and skills to perform cardiopulmonary resuscitation (CPR), as one never knows when they will be required. Studies have shown that a loss of skills occurs as early as 30 days after the training course, with variations according to personal and professional characteristics.

**Objectives:**

to assess whether medical students are able to retain skills acquired in a BLS course for more than six months.

**Methods:**

Prospective, case-control, observational study. Medical students attended a 40-hour course on sudden death and cardiac arrest. Skills acquired during the course were evaluated immediately after and six months after the course. Students’ individual scores were compared between these time points, the percentage of correct answers was evaluated, and overall performance was rated as excellent, good, and poor. Observers and evaluation criteria were the same immediately after the course and six months later. Data were analyzed using the paired t-test and the McNemar test. The 95% confidence interval was established, and a p < 0.05 was set as statistically significant.

**Results:**

Fifty students (27 female) in the first year of medical school aged from 18 to 24 years (mean of 21 years) attended the course. The number of steps successfully completed by the students at six months was significantly lower than immediately after the course (10.8 vs 12.5 p < 0.001). Neither sex nor age affected the results. Overall performance of 78% of the students was considered excellent immediately after the course, and this percentage was significantly higher than six months later (p < 0.01). After six months, the steps that the students failed to complete at six months were those related to practical skills (such as a correct hand positioning).

**Conclusion:**

A significant loss of skills was detected six months after the BLS course among medical students, compromising their overall performance.

## Introduction

Ischemic heart diseases are the main causes of death from cardiovascular disease,^1^ sudden death (SD), and cardiorespiratory arrest (CRA)^2^ in the Brazilian population.^1^ CRA may be reversed by cardiopulmonary resuscitation (CPR) maneuvers, and reduction of mortality and sequelae of CRA depends on an effective and fast intervention, started as soon as possible, preferably at the scene.^2 , 3^

Basic life support (BLS) is a series of procedures that can be initiated outside the hospital seting.^4 , 5^ A BLS course addresses CPR techniques that include early recognition of cardiac arrest and initiation of chest compression and ventilation maneuvers, and use of automated external defibrillator (AED), and is open to the lay public.^5 - 7^ Since most of CRA occurs out of hospital, it is important that CPR techniques can be performed by the general population,^8 - 10^ despite the common belief that only healthcare providers are able to act it properly in emergency situations.

However, an adequate training only is not enough. Rescuers should not only acquire but retain knowledge and skills for appropriate performance of CPR, as one never knows when they will have to be applied. Several studies have been conducted to assess the acquisition of these information and skills over time.^11 , 12^ Nevertheless, there is no consensus on what causes knowledge reduction and when it occurs, which makes it difficult, for example, to establish the frequency of retraining.

Studies have suggested a loss of ability to perform CPR even among healthcare providers, and possible causes include insufficient training and/or poor skill retention.^13^ Even these professionals may spend a long time without applying this knowledge. Smith et al.^11^ have shown that CPR psychomotor skills degrade within 10 weeks in nursing undergraduate students, and other studies have pointed out that practical skills start to decay as early as 30 days and continue up to one year after an advanced life support (ALS) course.^14^ A study published in 2014 reported a decrease in skill retention in undergraduate medical students, one and two years after a CPR course.^15 , 16^

As any person, medical students may witness SD or CRA events out of hospital settings, which would already warrant training on CPR maneuvers.^17 , 18^ Since the first months of college, medical students are expected to act as physicians and to have the same skills as professional ones.^19^ For this reason, we advocate that training on the management of CRA should be part of the medical school curriculum,^12^ with the BLS offered in their first undergraduate year, and the ALS at the end of the medical course, when the student will have acquired more knowledge and skills, and will be soon provide care to people. In our opinion, the guidelines published by the American Heart Association for CPR (e.g. BLS and Heart Saver) and by the Brazilian Society of Cardiology for cardiovascular emergencies (TECA B) meet the needs of healthcare providers as well as the lay population. However, for first-year medical school students, the basic course should include not only the training of skills, but also provide a wider theoretical basis, to facilitate the learning process and retention of abilities.^12 , 17^

Our hypothesis is that loss of knowledge and skills to successfully perform CPR maneuvers already occurs at six months after a BLS training among medical students, even in case of more detailed and longer courses.

## Methods

Fifty medical students (27 female) of the first year of college, aged between 18 and 24 years (mean of 21 years), attended the course entitled “Sudden death and cardiopulmonary resuscitation”. This is an optional course of the undergraduate curriculum, with a duration of 32 hours (30% theoretical classes and 70% practical classes), and emphasis on the development of skills and simulations of SD and/or CRA.

In addition to a theoretical content of history, epidemiology and pathophysiology of SD, this course also includes a practical content, where the students are trained to respond to a CRA event. During practical classes, students are trained to recognize and to respond to the signs of CRA – to check the safety of the scene, to know who and how to call for help, to perform effective chest compression (strength, rate, depth, site, and hand positioning), to perform safe and effective ventilation using the appropriate device, to verify the need for an AED and to learn how to use it, and to decide to either continue or interrupt the intervention. This practical training is based on the AHA (BLS)^16^ and the Brazilian Society of Cardiology (TECA B)^5^ guidelines for standardization of the management of CRA.

After the course, the knowledge and skills acquired by the students were assessed in an out-of-hospital setting using a standard form containing a checklist of procedures ( Chart 1 ).

**Chart 1:** – Checklist used to assess knowledge and skills acquired during the course “Sudden death and cardiopulmonary resuscitation” immediately after and six months after the course

PROCEDURES TO BE PERFORMED AFTER IDENTIFYING AN unconscious person WHO DOES NOT MOVE OR BREATHE
Check the safety of the scene and safety for intervention
Ask help to another person and state what must be done in a clear and objective way
Call 192 or 193
Request an automated external defibrillator (AED)
Position the victim correctly to check pulse and breathing
Initiate chest compressions immediately
Position the hands on the sternum
Perform chest compression with an adequate depth (4-5 cm)
Compress the chest at an adequate rate (100 per minute)
Perform ventilation only with a protection device
Perform five cycles of 30 compressions and two rescue breaths before reassessing the victim
Do not interrupt cardiopulmonary resuscitation (CPR) when an AED arrives
Use the AED following manufacturer’s instructions
Resume CPR after defibrillation if necessary

Each step was considered as successfully performed or not (YES if performed correctly or NO if not performed or if performed incorrectly). Then, overall performance was rated as excellent, good, or poor. An excellent performance was considered when up to two mistakes were made (which would be more than 84% of correct responses recommended by the AHA^16^ ); a good performance was considered when three or four mistakes were made (more than 70% of correct responses); and a poor performance when more than four mistakes were made (less than 70% of correct responses). To be approved in the course, the student had to give more than 70% of correct responses. Six months later, the students were reassessed using a CPR simulation mannequin, with no previous scheduling, and the same checklist and criteria (including the same instructors and teachers) used in the first assessment. Overall performance was rated also.

All students signed an informed consent form and agreed to participate in the study. The study, approval by the ethics committee and the consent form were approved and registered on Brazilian national ethics platform (Plataforma Brasil; CAAE: 81721317.7.0000.0082).

## Statistical analysis

This was a prospective study, where each patient was the control of him/herself. Results of the two assessments of the students’ performance were compared by the McNemar test for categorical variables (presented as absolute numbers and percentages). Means and standard deviations of continuous variables with normal distribution (tested by the Shapiro-Wilk test) were compared by paired t test using Microsoft Excel (Office 365). A 95% confidence level was calculated and a p-value lower than 0.05 was set as statistically significant.

## Results

All students were evaluated right after the course and six months thereafter. Of the 14 steps assessed, an average of 12.5 steps were correctly performed by the students immediately after the course, which was significantly higher than the average of 10.8 steps at six months (p < 0.001). Thus, there was a decay of knowledge and skills after six months of the course. In addition, although men had a higher mean age than women (21.7 years vs 20.2 years – p = 0.006), age and sex did not independently affect the results of the evaluations. The number of correct answers in the second evaluation was significantly lower as compared with the first evaluation in both men (10.9 vs. 12.8 correct answers; p = 0.003) and women (12.2 vs. 10.7 correct answers; p = 0.013). Regarding overall performance, the quality of care provided also decreased; while 39 (78%) students had an excellent performance in the first evaluation, performance of only 20 (40%) students was rated as excellent six months after (p < 0.01). The percentages of students who gave correct answers in each step of the checklist are described in Table 1 and Figure 1 .

**Table 1:** – Results of the assessment of students’ knowledge and skills acquired in the “Sudden death and cardiopulmonary resuscitation” course by numbers and percentages of students who successfully completed each step, immediately after and six months after the course

Step	Percentage of students who successfully completed the step		P*

	Immediate after (N)	At six months (N)	
1- Check the safety	42 (21)	54 (27)	0.307
2- Ask for help	88 (44)	84 (42)	0.683
3- Decide who to call	94 (47)	68 (34)	**0.002**
4- Request an automated external defibrillator	92 (46)	80 (40)	0.181
5- Check pulse and breathing	96 (48)	92 (46)	0.683
6 - Initiate chest compressions	100 (50)	94 (47)	0.248
7 - Position the hands correctly	90 (45)	66 (33)	**0.010**
8 - Correct depth of compression	96 (48)	88 (44)	0.289
9- Compression at an adequate rate	82 (41)	70 (35)	0.264
10- Perform ventilation with a protection device	88 (44)	62 (31)	**0.010**
11-Use the 30:2 compression-ventilation ratio	100 (50)	76 (38)	**0.002**
12-Do not interrupt cardiopulmonary resuscitation when the AED arrives	100 (50)	88 (44)	**0.041**
13-Use AED following manufacturer’s instructions	88(44)	80 (40)	0.387
14-Resume CPR after defibrillation if necessary	92(46)	84 (42)	0.387

* McNemar test (two-tailed); CPR: cardiopulmonary resuscitation; AED: automated external defibrillator.

**Figure 1:** – *Percentage of students that successfully completed the cardiac arrest procedures immediately after and six months after the course and six. Numbers correspond to those used in Table 1.*

## Discussion

Our study showed a significant reduction (p < 0.01) in skills related to the management of CRA. The students correctly completed a mean of 12.5 steps immediately after the course and 10.8 steps six months later. While 39 (78%) students successfully completed more than 12 of the 14 steps of the simulated cardiac arrest event immediately after the course, only 20 (40%) achieved the same score six months later.

We evaluated the skills required for an adequate and correct intervention of CRA and observed that the students successfully acquired them at the end of the course. The first step (where students had to check safety before intervening) was the only step that was not correctly performed by most of the students (42%). When questioned about it, the students explained that, since simulation was carried out in a classroom, that is known to be safe, and using a mannequin, they have forgotten to check safety. Another explanation was the eager to rapidly initiate the intervention. Despite that, the outcome of the course was considered satisfactory, according to the AHA recommendations of a minimum of 84% of correct answers.^16^ We also found that neither age nor sex influenced the students’ score in the end of the course, reinforcing the importance of the course in knowledge acquisition.

One common problem related to teaching the management of CRA events is how to retain knowledge. A meta-analysis^14^ on the theme made it clear that skills become lost when they are not practiced, initiating within few weeks, achieving a maximum at approximately nine months to one year. These studies have shown that the time elapsed from the last training is directly proportional to the degree of loss of the skills and knowledge needed to deliver CPR effectively.^14^

In our study, such decay in skills and knowledge was also observed in first-year medical students, with a loss of approximately 15% in the test of skills, which caused a loss of nearly 50% in overall performance. We did not expect that the loss of knowledge retention was different among medical students, since previous studies have already shown that with healthcare professionals and students.^13^ Also, we do know that students do not frequently practice managing CRA events. In fact, a study comparing knowledge loss between medical students and other students is warranted.

In our sample, there was a clear loss of skills, especially in the practical steps that require more attention and promptness. This is corroborated by previous studies reporting that these steps were more frequently forgotten or inadequately performed.^21 , 22^ When these practical steps are not properly performed (decide who to call; position and place hands correctly, perform ventilation with protection device, continue performing the five cycles and the maneuvers when an AED arrives) the risk for adverse events or complications increases, such as rib fractures and ineffective compressions, resulting in ineffective circulation.^23^

Also, neither age nor sex affected retention of knowledge and skills by our students, and time seems to be the only factor responsible for retention loss. This is reinforced by the fact that none of the students responded to CRA events or helped teaching CPR courses.

One strategy to retain skills, proposed in previous studies,^17 , 24^ is to participate in continuous training, such as the e-learning. However, one question still unanswered is how often and what type of training would be recommended and who is the training for (healthcare professionals or lay people, who work in different settings where CRA events are relatively common). Another question is whether the course should be more comprehensive or maybe simpler, particularly for medical students, which would be answered by a comparative study between the two methods.

One limitation of the study is the small number of students evaluated and the fact that, despite its prospective design, the study was conducted in a single center, and after one training course. Ruijter et al.^15^ also demonstrated a reduction in retention of skills after one and two years of BLS training in 120 medical students, similar to our findings. Therefore, although the aim of the study was to assess skill retention, our results were in accordance with previous studies, and this limitation does not affect the importance of our study. Another limitation for a more in-depth discussion about skill retention and of what is the most appropriate type of course is the fact that we did not evaluate the theoretical knowledge of students. Some studies, however, have already shown that more important losses have occurred in practical skills.^11^ Although the students knew that they would be reevaluated at some point after the course, this seemed not to have affected the results; many of them have forgotten this reevaluation as they had already been approved in the course. However, this fact does not undermine the need for more frequent training courses to try to reduce the loss of skills.

## Conclusion

Six months after a BLS course in simulated SD or CRA events, we observed a significant loss of skills in first-year medical students, similarly to what is observed in the lay population (outside the healthcare filed). This loss was associated with the period during which the students did not practice or review the techniques to correct perform CPR, which affects treatment efficacy. A more robust course may improve learning, but not the retention of skills. Finally, the assessment of learning and knowledge retention could be complemented by studies including retraining and clinical outcomes, i.e., aiming to demonstrate that training and retraining may not only improve skills but also save lives.
